# 9-Methylfascaplysin, a Marine-Derived Bioactive Compound, Promotes Neurite Outgrowth via the Inhibition of ROCK2

**DOI:** 10.3390/ph18111751

**Published:** 2025-11-17

**Authors:** Meilin Zheng, Kangyang Gao, Yirui Hong, Jingyang Le, Jingjing Cai, Hongze Liang, Wei Cui

**Affiliations:** 1 Translational Medicine Center of Pain, Emotion and Cognition, Health Science Center, Ningbo University, Ningbo 315211, China; linz9930@163.com (M.Z.);; 2 School of Materials Science and Chemical Engineering, Ningbo University, Ningbo 315211, China; 3 The Affiliated Kangning Hospital of Ningbo University, Ningbo University, Ningbo 315201, China

**Keywords:** 9-methylfascaplysin, ROCK2, neurite outgrowth, GAP-43, neurodegenerative disorders, marine bioactivity

## Abstract

**Background:** The impairment of neurite outgrowth is an early pathological hallmark underlying various neurodegenerative disorders. The promotion of neurite outgrowth was considered as a feasible strategy to treat neurodegenerative disorders. 9-Methylfascaplysin (9-MF), a marine-derived, bioactive compound, has exhibited multiple neuroprotective activities. **Methods and Result:** In this study, 9-MF at nanomolar concentrations promoted neurite outgrowth, upregulated the expression of growth-associated protein-43 (GAP-43), and increased the mitochondrial positive area with similar efficacy as retinoic acid in PC12 cells. 9-MF-associated differentiated expressed genes were enriched in mitochondria and synapse, forming a Rho-associated coiled-coil containing a protein kinase 2 (ROCK2)-centralized network. CMap analysis further identified positive connections between 9-MF-induced perturbation and perturbations caused by the inhibition of the ROCK2 pathway. Molecular docking analysis demonstrated a high binding affinity between 9-MF and ROCK2, indicating that 9-MF could inhibit ROCK2. Furthermore, 9-MF significantly reduced the phosphorylation of ROCK2 with a similar efficacy as fasudil, a ROCK2 inhibitor. Narciclasine, a known ROCK2 activator, almost completely abolished the effects of 9-MF on the induction of neurite outgrowth in PC12 cells. **Conclusions:** 9-MF effectively promoted neurite outgrowth possibly via the inhibition of ROCK2, providing supporting evidence that 9-MF might be developed as a novel neurological drug.

## 1. Introduction

9-Methylfascaplysin (9-MF) is a marine-derived bioactive compound synthesized based on fascaplysin, a benzoyl-linked β-carboline alkaloid isolated from the *Fijian sponge Fascaplysinopsis* sp. [1,2]. 9-MF could produce broad-spectrum neuroprotectivity, including the inhibition of the β-amyloid (Aβ) oligomer formation, the reduction in acetylcholinesterase activity, and the prevention of H_2_O_2_- and Aβ-induced synaptic and neuronal toxicity [3]. In addition, 9-MF inhibited middle cerebral artery occlusion/reperfusion-induced oxidative stress and the activation of nuclear factor κB and NLRP3 inflammasome [4]. Our recent study showed that 9-MF prevented neuroinflammation and synaptic damage in APP/PS1 transgenic mice [5]. Moreover, after a single intragastric administration, 9-MF could be retained in the brain, suggesting that 9-MF might be developed as a promising marine-inspired neuroprotective agent to treat neurological disease [6].

The impairment of neurite outgrowth is an early pathological hallmark underlying multiple neurodegenerative disorders, including Alzheimer’s disease (AD), Parkinson’s disease, and ischemic stroke [7]. In these disorders, neurite degeneration normally occurs prior to neuronal loss and behavioral impairments, and the regenerative potential of impaired neurites is inhibited by the microenvironment, including neuroinflammation and oxidative stress within the brain [8]. Therefore, it is widely accepted that the therapeutic promotion of neurite outgrowth is a feasible strategy for the treatment of neurodegenerative disorders.

PC12 cells offer several advantages as in vitro models for neurite outgrowth, including their ease of cultivation and the well-characterized mechanisms underlying neurite outgrowth. Therefore, PC12 cells have been widely employed as screening pharmacological agents for the promotion of neurite outgrowth [9]. Rho-associated coiled-coil containing protein kinase 2 (ROCK2) serves as a pivotal kinase in the Rho/ROCK signaling pathway, participating in diverse cellular functions, including cell morphology, motility, survival, apoptosis, cytoskeletal regulation, and neurite outgrowth [10]. The activation of ROCK2 could be regulated by Rho guanine nucleotide exchange factors and Rho GTPase-activating proteins, leading to the formation of GDP- and GTP-bound forms of RhoA, respectively [11]. ROCK2 could further phosphorylate its downstream targets, including myosin light chain and LIM-domain-containing protein kinase (LIMK), leading to the excessive stabilization of the actin cytoskeleton, inhibiting growth cone navigation, axonal extension, and neurite outgrowth [12]. Notably, fasudil, a classical ROCK2 inhibitor used in the treatment of cerebral vasospasm, could promote neurite outgrowth and enhance synaptic plasticity, suggesting that ROCK2 inhibitors might be developed as promoters of neurite outgrowth [13].

In this study, we have evaluated the neurite outgrowth promotion effects of the marine-derived compound 9-MF in PC12 cells, in compassion to retinoic acid, a well-known promoter of neurite outgrowth. The molecular mechanisms underlying the neurite outgrowth promotion effects of 9-MF were further elucidated by using high-content imaging, RNA-seq analysis, bioinformatical analysis, Western blotting analysis, and immunocytochemical staining, and confirmed by using ROCK2-related tool drugs.

## 2. Results

### 2.1. Subsection

#### 2.1.1. 9-MF Induces Neurite Outgrowth in PC12 Cells

**Figure 1:**
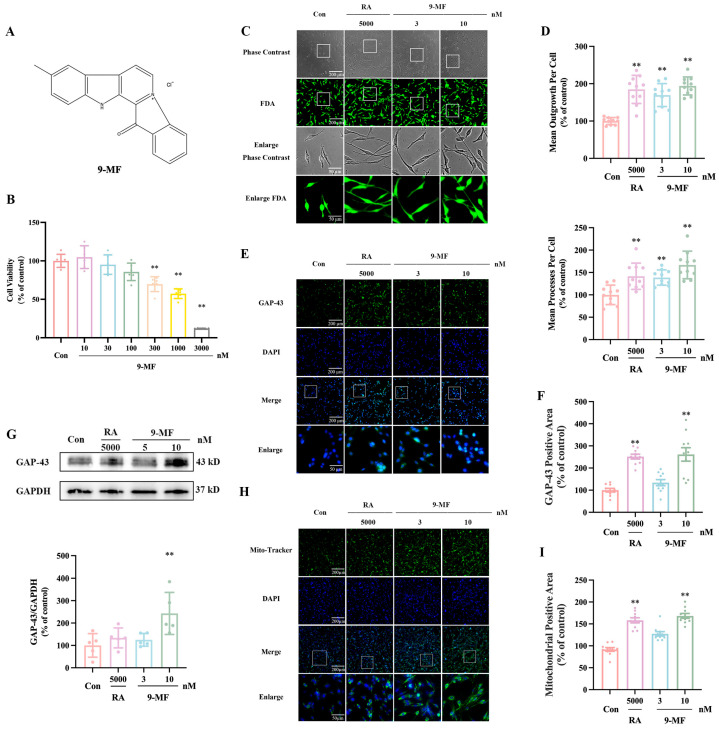
9-MF induces neurite outgrowth in PC12 cells. (**A**) The chemical structure of 9-MF. 9-MF at various concentrations was added into PC12 cells for 24 h. (**B**) Cell counting kit8 (CCK8) assay was employed to measure cell viability (n = 6). Retinoic acid and 9-MF were added into PC12 cells, respectively, for 48 h. (**C**) FDA staining was employed to measure mean outgrowth and mean processes per cell. (**D**) The quantitative results of (**C**) showed that 9-MF induced neurite outgrowth in PC12 cells (n = 10). (**E**) Immunocytochemical staining was employed to measure GAP-43-positive area. (**F**) The quantitative results of (**E**) showed that 9-MF upregulated GAP-43-positive area in PC12 cells (n = 10). (**G**) Western blotting analysis was employed to measure the expression of GAP-43 and GAPDH (n = 5). (**H**) Mito Tracker staining was employed to measure the mitochondrial positive area. (**I**) The quantitative results of (**H**) showed that 9-MF increased the mitochondrial positive area in PC12 cells (n = 10). RA: retinoic acid. Data were presented as the mean ± SD. ** *p* < 0.01 vs. the control group (one-way ANOVA and Tukey’s test).

The chemical structure of 9-MF is demonstrated in Figure 1A. 9-MF, at various concentrations, was added into PC12 cells. After 24 h, 10–30 nM 9-MF did not significantly induce neurotoxicity in PC12 cells, indicating the safety of 9-MF below these concentrations (Figure 1B). To investigate the effects of 9-MF on the promotion of neurite outgrowth, 3–10 nM 9-MF were administrated into PC12 cells. In the same condition, 5 μM retinoic acid was used as a positive control. After 48 h, the morphology of outgrowth neurites and the expression of GAP-43 were evaluated. Both 9-MF and retinoic acid significantly increased neurite outgrowth, GAP-43-positive area, and GAP-43 expression in PC12 cells (Figure 1C–G). In addition, the neurite outgrowth promotion effects of 10 nM 9-MF were comparable to those of 5 μM retinoic acid (Figure 1C–G). Furthermore, an increase in mitochondrial positive area was observed in 9-MF- and retinoic-acid-treated PC12 cells, respectively (Figure 1H,I). Moreover, 10 nM 9-MF increased mitochondrial positive area, in a manner comparable to that of 5 μM retinoic acid (Figure 1H,I). These results suggested that 9-MF induced neurite outgrowth with similar efficacy to retinoic acid in PC12 cells.

#### 2.1.2. 9-MF Induces Neurite Outgrowth via the Action on a ROCK2-Centralized Network

**Figure 2:**
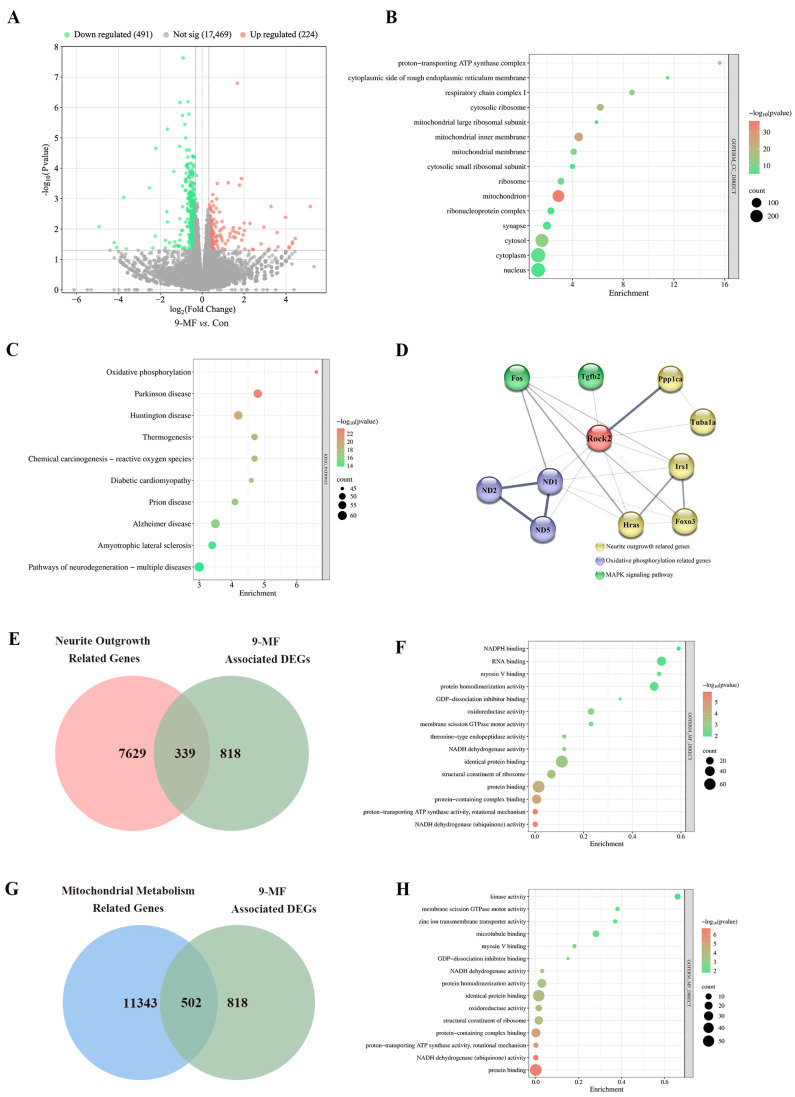
9-MF induces neurite outgrowth via the action on a ROCK2-centralized network. (**A**) Volcano plot showing RNA-seq results from 9-MF- and vehicle-treated PC12 cells. (**B**) GO-CC analysis of 9-MF-associated DEGs showing the enrichment in mitochondria and synapse. (**C**) KEGG analysis of 9-MF-associated DEGs showing enrichment in various neurodegenerative disorders. (**D**) The PPI network of 9-MF-associated DEGs showing a ROCK2-centralized network. (**E**) Venn diagram showing the intersection of 9-MF-associated DEGs and neurite-outgrowth-related genes. (**F**) GO-MF of DEGs in (**E**) showing the enrichment in kinase activity and microtubule binding. (**G**) Venn diagram showing the intersection of 9-MF-associated DEGs and mitochondrial metabolism-related genes. (**H**) GO-MF of DEGs in (**G**) showing the enrichment of DEGs in GDP-dissociated inhibitor binding and GTPase motor activity.

We further explored the molecular mechanisms underlying the effects of 9-MF on the promotion of neurite outgrowth. Total mRNA from 9-MF- and vehicle-treated PC12 cells was extracted after screening with the threshold of |log_2_(FC)| > 0.3 and *p* value < 0.05. In total, 715 9-MF-associated differentially expressed genes (DEGs), including 224 upregulated and 491 downregulated genes, were identified. The volcano plot of 9-MF-associated DEGs is demonstrated in Figure 2A. Gene Ontology Cellular Component (GO-CC) analysis of 9-MF-associated DEGs showed the enrichment in mitochondria and synapse, suggesting that 9-MF mainly regulates targets in mitochondria and synapse (Figure 2B). Kyoto Encyclopedia of Genes and Genomes (KEGG) analysis of 9-MF-associated DEGs showed the enrichment in various neurodegenerative disorders (Figure 2C). The string analysis data were imported into Cytoscape software (Version 3.10.3). Hub genes, including Rock2, Ppp1ca, Tuba1a, Tgfb2, Fos, ND1, ND2, ND5, Hras, Foxo3, and Irs1, were identified. The protein–protein interaction (PPI) network was generated by the interactions among these hub genes, demonstrating a ROCK2-centralized network (Figure 2D). Venn plot was further established, which showed that 339 genes were within 9-MF-associated DEGs and neurite-outgrowth-related genes (Figure 2E). Gene Ontology Molecular Function (GO-MF) analysis of these genes showed the enrichment in kinase activity and microtubule binding (Figure 2F). A Venn diagram is demonstrated in Figure 2G, showing the intersection of 9-MF-associated DEGs and mitochondrial metabolism-related genes. GO-MF analysis of these genes showed that the enrichment in GDP-dissociated inhibitor binding and GTPase motor activity (Figure 2H).

#### 2.1.3. 9-MF Inhibits ROCK2

**Figure 3:**
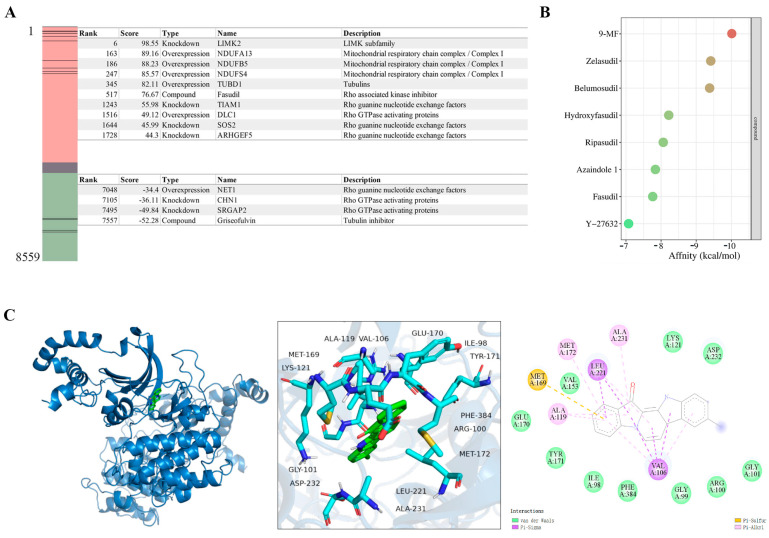
9-MF inhibits ROCK2. (**A**) CMap analysis revealed that the transcriptional response to 9-MF positively correlated with ROCK2 pathway inhibition, including profiles induced by LIMK2 knockdown and Rho GTPase-activating protein overexpression. Conversely, 9-MF negatively correlated with perturbations associated with Rho pathway activation. (**B**) Dot plot showing the affinities between various ROCK2 inhibitors and ROCK2. The size and color of the dots correspond to the affinity. The specific affinities between ROCK2 and inhibitors are as follows: 9-MF (−10.0 kcal/mol), zelasudil (−9.41 kcal/mol), belumosudil (−9.38 kcal/mol), hydroxyfasudil (−8.22 kcal/mol), ripasudil (−8.06 kcal/mol), azaindole-1 (−7.84 kcal/mol), fasudil (−7.81 kcal/mol), and Y-27632 (−7.07 kcal/mol). (**C**) The best conformation between 9-MF and ROCK2 was elucidated by molecular docking analysis.

The potential targets of 9-MF were predicted by Connectivity Map (CMap) screening, using 9-MF-associated DEGs. ROCK2 could directly phosphorylate LIMK [14]. In addition, the activity of ROCK2 could be inhibited by Rho GTPase activating proteins, and promoted by Rho guanine nucleotide exchange factors [15]. CMap analysis revealed that the transcriptional response to 9-MF positively correlated with ROCK2 pathway inhibition, including profiles induced by LIMK2 knockdown and Rho GTPase-activating protein overexpression. Conversely, 9-MF negatively correlated with perturbations associated with Rho pathway activation (Figure 3A). These results suggested that 9-MF might inhibit the ROCK2 pathway, which is consistent with our previous study showing that 9-MF could inhibit the kinase activity of ROCK2 [5].

Molecular docking analysis was used to evaluate the affinities between ROCK2 and ROCK2 inhibitors. Zelasudil, belumosudil, hydroxyfasudil, ripasudil, azaindole 1, fasudil, and Y-27632, seven known ROCK2 inhibitors, exhibited the affinities of −9.41, −9.38, −8.22, −8.06, −7.84, −7.81, and −7.07 kcal/mol, respectively, for the interactions with ROCK2 (Figure 3B). At the same condition, 9-MF demonstrated an optimal binding affinity of −10.0 kcal/mol for ROCK2 (Figure 3B). Docking conformation analysis revealed that 9-MF engaged in van der Waals with ILE98, GLY99, ARG100, GLY101, LYS121, VAL153, GLU170, TYR171, ASP232, and PHE384, and hydrophobic interactions with ALA119, MET172, and ALA231 within ROCK2. In addition, π–σ interactions were predicted between 9-MF, and VAL106 and LEU221, and a π–sulfur contact was predicted between 9-MF and MET169 within ROCK2 (Figure 3C).

#### 2.1.4. 9-MF Induces Neurite Outgrowth via the Inhibition of ROCK2

**Figure 4:**
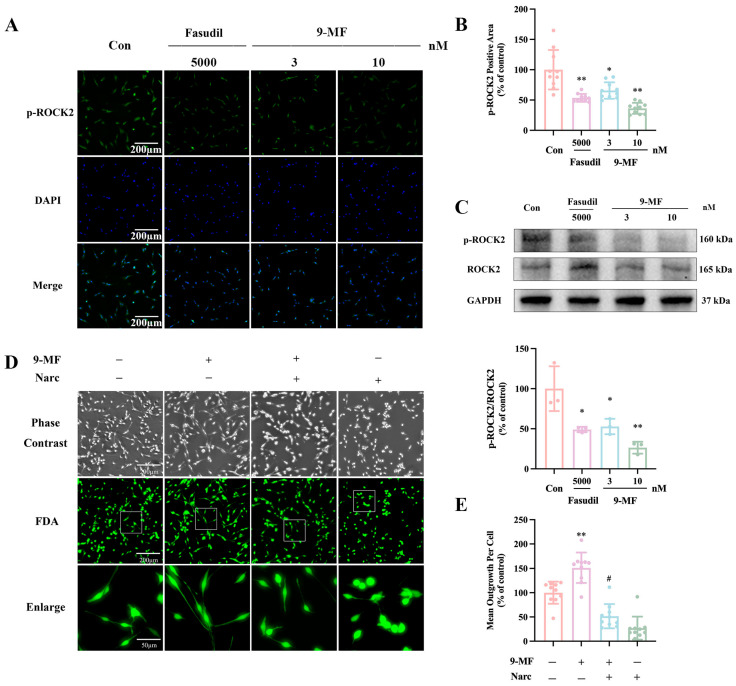
9-MF induces neurite outgrowth via the inhibition of ROCK2. 9-MF and fasudil were added into PC12 cells for 0.5 h. (**A**) Immunocytochemical staining was employed to measure p-ROCK2-positive area. (**B**) The quantitative results of (**A**) showed that 9-MF upregulated the p-ROCK2-positive area in PC12 cells (n = 10). (**C**) Western blotting analysis was employed to measure the expression of p-ROCK2 and ROCK2 (n = 3). 9-MF and narciclasine were added into PC12 cells for 48 h. (**D**) FDA staining was employed to measure neurite outgrowth. (**E**) The quantitative results of (**D**) showed that 9-MF induced neurite outgrowth in PC12 cells (n = 10). Narc: narciclasine. Data were presented as the mean ± SD. * *p* < 0.05 and ** *p* < 0.01 vs. the control group in (**B**,**C**) (one-way ANOVA and Tukey’s test), ** *p* < 0.01 vs. the control group, and ^#^ *p* < 0.05 vs. the 9-MF group in (**E**) (two-way ANOVA and Tukey’s test).

Fasudil is a well-characterized ROCK2 inhibitor. 9-MF (3–10 nM) and fasudil (5 μM) were administrated into PC12 cells. After 0.5 h, the expression of p-ROCK2 was evaluated. Both 9-MF and fasudil significantly decreased the p-ROCK2-positive area in PC12 cells (Figure 4A,B). The ROCK2 inhibition effects of 10 nM 9-MF were comparable to that of the 5 μM fasudil (Figure 4A,B). The inhibition of 9-MF on ROCK2 phosphorylation was further confirmed by Western bolting analysis (Figure 4C). Narciclasine is a known ROCK2 activator [16]. Narciclasine effectively counteracted the neurite outgrowth promotion effects of 9-MF in PC12 cells, supporting the idea that 9-MF promoted neurite outgrowth via the inhibition of ROCK2 (Figure 4D,E).

## 3. Discussion

In this study, we have demonstrated that 9-MF effectively promoted neurite outgrowth with similar efficacy and higher potency than retinoic acid, possibly via the inhibition of ROCK2, in PC12 cells.

PC12 cells are widely used to screen agents with neurite outgrowth promotion activity. Panaxynol, a neuroprotective agent extracted from Panax ginseng, could promote neurite outgrowth by activating the cAMP-CREB axis in PC12 cells [17]. Mottenohoka, a compound derived from dendranthema and grandiflorum cv., could enhance neurite outgrowth by suppressing p38MAPK phosphorylation in PC12 cells [18]. 9-MF produced neurite outgrowth promotion effects at nanomolar concentrations, ranking it among the most potent promoters of neurite outgrowth. To date, 9-MF is about 500 times more potent than retinoic acid, a classic promoter of neurite outgrowth, in inducing neurite outgrowth in PC12 cells.

Neurite outgrowth could be characterized from multiple aspects of biological processes, including the increased neurite number and length, the elevated expression of neurite-associated proteins, and the increase of ATP production [19]. In our study, 9-MF not only increased the length of neurites, but also upregulated the expression of GAP-43, a neurite-associated protein, and promoted mitochondrial positive area in PC12 cells. These results indicated that 9-MF promoted neurite outgrowth via the action on neurite-associated and mitochondria-related proteins simultaneously. This speculation was further supported by the RNA-seq data, showing that 9-MF-associated DEGs were mainly enriched in mitochondria and synapse.

How could 9-MF promote neurite outgrowth in PC12 cells? The inhibition of ROCK2 has been reported to promote mitochondrial metabolism and regulate the cytoskeleton, leading to the induction of neuronal differentiation and neurite outgrowth [20]. 1,3-benzodioxol-5-yl [1-(5-isoquinolinylmethyl)-3-piperidinyl]-methanone, a ROCK2 inhibitor, could significantly promote neurite outgrowth [21]. DNA topoisomerase IIβ could induce neurite extension in human mesenchymal stem cells via the modulation of the ROCK2 pathway [22]. Qiangji decoction could upregulate ATP production via the downregulation of ROCK2, thereby stimulating neurite outgrowth and exerting neuroprotective effects [23]. These findings collectively supported that 9-MF might promote neurite outgrowth via the action on ROCK2. PPI analysis of 9-MF-associated DEGs suggested that 9-MF could act on a ROCK2-centralized network. CMap analysis further suggested positive connections between 9-MF-induced perturbation and perturbations caused by the inhibition of the ROCK2 pathway, and suggested negative connections between 9-MF-induced perturbation and perturbations caused by the activation of the ROCK2 pathway. Most importantly, our previous study has demonstrated that 9-MF could inhibit the kinase activity of ROCK2, which was confirmed by an in vitro kinase activity assay [5]. Molecular docking analysis further showed that 9-MF interacted with ROCK2 with van der Waals force, alkyl–alkyl interactions, π–σ interactions, and π–sulfur contacts. In addition, 9-MF was predicted to possess the highest binding affinity to ROCK2 among various known ROCK2 inhibitors. Furthermore, 9-MF significantly reduced the phosphorylation of ROCK2 with similar efficacy to fasudil. Narciclasine almost completely abolished the effects of 9-MF on the induction of neurite outgrowth. These results suggested that 9-MF might induce neurite outgrowth by inhibiting ROCK2. Although we have previously discovered that 9-MF could inhibit ROCK2 in microglial cells [5], the effects of 9-MF on neuronal ROCK2 were not reported previously. Our current work fills this critical knowledge gap by demonstrating 9-MF-mediated ROCK2 inhibition in PC12 cells, presenting a significant advancement in understanding the cell-type-specific mechanism of 9-MF.

Neurite outgrowth not only represents a fundamental process in neuronal development, but also plays a pivotal role in neuroplasticity and neuronal repair [24]. The impairments of neurite growth could be found during the onset and the progression of neurodegenerative disorders, with structurally and functionally abnormal neurites appearing prior to neuronal loss [25]. Therefore, the maintenance of neurite integrity and the restoration of neurite growth capacity have emerged as promising therapeutic strategies for treating neurodegenerative diseases. 9-MF was reported to produce anti-AD and anti-ischemic stroke neuroprotection effects [4]. In this study, 9-MF was proven to be a potent promoter of neurite outgrowth, providing more evidence that this marine-derived bioactive compound could be developed as a novel neurological drug.

Our study has some limitations. Firstly, the neurite outgrowth promotion effects of 9-MF were not validated in vivo. We plan to evaluate the therapeutic effects of 9-MF in animal models. For example, neuronal tract-tracing techniques, as well as a cortical lesion animal model, could be used to evaluate whether 9-MF could enhance neurite outgrowth and regeneration in vivo. In addition, conditional ROCK2 knockout mice could be used to investigate whether the observed effects of 9-MF were specifically mediated through ROCK2 inhibition. Secondly, although 9-MF could induce neurite outgrowth via the inhibition of ROCK2 activity, it can still not be excluded that 9-MF promoted neurite outgrowth from ROCK2-independent pathways.

## 4. Materials and Methods

### 4.1. Chemicals

9-MF was synthesized according to a previous study by Dr. Xiao Zhiyong from Health BioMed Co., Ltd. (Ningbo, China) [3]. 9-MF was further dissolved in H_2_O with 0.1% dimethyl sulfoxide (DMSO). Retinoic acid and fasudil were purchased from Aladdin Biochemical Technology Co. (Shanghai, China). Narciclasine was purchased from Med Chem Express (Rocky Hill, NJ, USA). All these agents were dissolved in H_2_O with 0.1% DMSO.

### 4.2. PC12 Cells

PC12 cells (Chinese Academy of Sciences, Shanghai, China) were cultured in Dulbecco’s modified Eagle’s medium (DMEM) supplemented with 10% heat-inactivated fetal bovine serum (FBS) and 1% penicillin/streptomycin with 5% CO_2_ at 37 °C. The cells were replaced with medium every 2 d. When the intensity of cell was observed to be around 80–90%, cells were seeded in 6-well or 96-well plates.

### 4.3. Cell Viability Assay

Before the addition of compounds, cells were switched to growth medium (DMEM supplemented with 0.5% FBS). Cell viability was evaluated using the cell counting kit 8 (CCK8, NCM Biotech, Newport, RI, USA). Then, 10 µL CCK8 was added into each well for 1 h with 5% CO_2_ at 37 °C. Optical density was measured at 450 nm using a microplate reader (Multiskan GO, Thermo Fisher Scientific, Waltham, MA, USA).

### 4.4. Fluorescein Diacetate (FDA) Staining

PC12 cells were treated with 9-MF at varying concentrations (3 and 10 µM) or with retinoic acid (5 µM) as a positive control. According to previous studies, retinoic acid at 5 μM could induce neurite outgrowth in PC12 cells [26,27]. Neurite outgrowth was analyzed using FDA staining, as previously described [28]. Cell imaging was conducted using a high-content ImageXPress Micro XL system (Molecular Devices, San Jose, CA, USA). Images were captured using a 20× objective lens. Image acquisition for FDA was detected using the FITC filter. Images were processed and analyzed using the Neurite Outgrowth module of MetaXpress software (Molecular Devices, Version 6.7.2.290). This module automatically identified cell bodies and neurites from images of FDA-stained cells and calculated the average neurite length per cell across the entire field of view. This process eliminated observer bias and ensured consistent measurement criteria across all experimental groups [29].

### 4.5. Mitochondrial Staining

Mitochondrial staining was performed using MitoTracker™ Green FM (Thermo Fisher Scientific). Cells were incubated with the dye for 30 min with 5% CO_2_ at 37 °C. Cells were then washed three times with pre-warmed PBS and stained with 4′,6-diamidino-2-phenylindole (DAPI) to visualize the nuclei. The cell imaging was conducted using a high-content ImageXPress Micro XL system. The images were captured using a 20× objective lens. Image acquisition for MitoTracker™ Green FM was detected using the FITC filter. Image acquisition for DAPI was detected using DAPI filter. Images were processed and analyzed using the custom module of the MetaXpress software (Molecular Devices, Version 6.7.2.290).

### 4.6. RNA-Seq

PC12 cells in 9-MF- and vehicle-treated groups were homogenized using TRIzol reagent (Omega Bio-Tek, Norcross, GA, USA) at 4 °C. RNA-Seq was performed and processed with a previously reported protocol [30]. To avoid missing the subtle but coordinated important regulatory events, genes with a *p*-value less than 0.05 and absolute log_2_|FC| greater than 0.3 were considered as DEGs. Human genome sequences and gene annotations were obtained from the University of California Santa Cruz Genome Website (http://genome.ucsc.edu/). Only the coding transcripts were included in the study.

### 4.7. Bioinformatical Analysis

A total of 7629 neurite-outgrowth-related genes and 11,343 mitochondrial metabolism-related genes were download from GeneCards database (https://www.genecards.org/, accessed on 16 July 2025). All genes were converted to Rattus norvegicus origin via g:Profiler (https://biit.cs.ut.ee/gprofiler/convert, accessed on 17 July 2025). GO enrichment analysis and pathway enrichment analysis were performed using David Bioinformatics Resources (https://davidbioinformatics.nih.gov/home.jsp, accessed on 18 July 2025). Bubble plots were generated using bioinformatics websites (https://www.bioinformatics.com.cn/, accessed on 18 July 2025). PPIs were analyzed using the online database STRING, and the PPI network was visualized through Cytoscape (version 3.10.3).

### 4.8. CMap Analysis

CMap analysis was conducted as previously described [31]. The top 150 upregulated and 150 downregulated genes were analyzed in the CMap database to identify potential small-molecule perturbations and therapeutic candidates. The perturbagens encompass 8559 signatures for perturbagen, indicating the relationship between the gene list and all perturbations in the CMap. The CMap score ranges from −99.39 to 99.82. A higher score indicates a higher likelihood of similar effects.

### 4.9. Molecular Docking Analysis

The high-resolution crystal structure of the ROCK2 (PDB ID: 6ED6) was screened using the RCSB PDB database (http://www.rcsb.org/, accessed on 3 September 2025). The chemical structure of 9-MF was processed using PyMOL (Version 3.1.6.1). Molecular docking was performed using AutoDock Vina 1.5.6 to investigate protein–ligand interactions. The docking grid box coordinate was determined to encompass the binding site. The optimal binding conformation was selected based on docking scores, and protein–ligand interactions were visualized using PyMOL.

### 4.10. Western Blotting Analysis

Western blotting assay was performed as previously described [32]. Briefly, cells were harvested and lysed. The proteins collected by centrifugation were separated on SDS-PAGE gel and then transferred to polyvinylidene fluoride membranes. The membranes were blocked with 5% bovine serum albumin in tris-buffered saline with Tween-20 (TBST) for 2 h and incubated overnight at 4 °C with primary antibodies [GAPDH (1:2000), GAP-43 (1:2000), ROCK2 (1:2000), or p-ROCK2 (Ser1366, 1:2000) were from Affinity Biosciences, Cincinnati, OH, USA]. After washing the samples three times with TBST, the membranes were incubated with anti-rabbit secondary HRP-linked antibodies (1:3000, Cell Signaling Technology, Danvers, MA, USA) for 1 h at room temperature. The membranes were rinsed by using TBST three times, then enhanced chemiluminescence substrate (Amersham Biosciences, Buckingham-shire, UK) was added. The analysis was performed using a fully automated chemiluminescence image analysis system (Tanon Science & Technology Co. Ltd., Shanghai, China). Protein expression was analyzed by using Image J (FIJI for Mac OS).

### 4.11. Immunocytochemical Staining

PC12 cells were fixed in 4% paraformaldehyde for 20 min. After blocking at room temperature in blocking buffer (1% bovine serum albumin and 0.2% Triton X-100) for 1 h, cells were exposed to the primary antibody [GAP-43 (1:500) or p-ROCK2 (1:500)] for 30 min, followed by the addition of the FITC-conjugated anti-rabbit secondary antibody (1:200, Abclonal, Wuhan, China) for 30 min. Cells were washed twice in PBS, and stained with DAPI to visualize the nuclei. The cell imaging was conducted using a high-content ImageXPress Micro XL system. Images were captured using a 20× objective. Image acquisition for GAP-43 or p-ROCK2 was detected using the FITC filter. Image acquisition for DAPI was detected using the DAPI filter. Images were processed and analyzed using the custom module of the MetaXpress software (Molecular Devices, Version 6.7.2.290).

### 4.12. Statistical Analysis

The results were expressed as mean ± standard derivation (SD). GraphPad Prism (version 10.0.0, Boston, MA, USA) was used to analyze the data. The differences in groups were evaluated by the *t*-test, one-way analysis of variance (ANOVA), or two-way ANOVA with Tukey’s test. *p* < 0.05 was regarded as statistically significant.

## 5. Conclusions

In conclusion, our study has demonstrated that 9-MF, a marine-derived bioactive compound, effectively promoted neurite outgrowth, possibly via the inhibition of ROCK2, providing evidential support that 9-MF could be developed as a novel neurological drug.

## Data Availability

The original contributions presented in this study are included in the article. Further inquiries can be directed to the corresponding author(s).

## Abbreviations

The following abbreviations are used in this manuscript: AD: Alzheimer’s disease; ANOVA: analysis of variance; Aβ: β-amyloid; CCK8: cell counting kit 8; CMap: Connectivity Map; DAPI: 4′,6-diamidino-2-phenylindole; DEGs: differentially expressed genes; DMEM: Dulbecco’s modified Eagle’s medium; FBS: fetal bovine serum; FDA: fluorescein diacetate; GAP-43: growth-associated protein-43; GO: Gene Ontology; LIMK: LIM domain-containing protein kinase; KEGG: Kyoto Encyclopedia of Genes and Genomes; PPI: protein–protein interactions; ROCK2: Rho-associated coiled-coil containing protein kinase 2; SD: standard derivation; TBST: tris-buffered saline with Tween-20; and 9-MF: 9-methylfascaplysin.
